# Influence of particle size on non-Darcy seepage of water and sediment in fractured rock

**DOI:** 10.1186/s40064-016-3778-9

**Published:** 2016-12-20

**Authors:** Yu Liu, Shuncai Li

**Affiliations:** 1 School of Mechanical and Electrical Engineering, Jiangsu Normal University, Xuzhou, 221116 Jiangsu China; 2 School of Mines, China University of Mining and Technology, Xuzhou, China

**Keywords:** Fracture, Water–sand, Non-Darcy seepage, Non-Newton fluid

## Abstract

Surface water, groundwater and sand can flow into mine goaf through the fractured rock, which often leads to water inrush and quicksand movement. It is important to study the mechanical properties of water and sand in excavations sites under different conditions and the influencing factors of the water and sand seepage system. The viscosity of water–sand mixtures under different particle sizes, different concentration was tested based on the relationship between the shear strain rate and the surface viscosity. Using the self-designed seepage circuit, we tested permeability of water and sand in fractured rock. The results showed that (1) effective fluidity is in 10^−8^–10^−5^ m^n+2^ s^2−n^/kg, while the non-Darcy coefficient ranges from 10^5^ to 10^8^ m^−1^ with the change of particle size of sand; (2) effective fluidity decreases as the particle size of sand increased; (3) the non-Darcy coefficient ranges from 10^5^ to 10^8^ m^−1^ depending on particle size and showed contrary results. Moreover, the relationship between effective fluidity and the particle size of sand is fitted by the exponential function. The relationship between the non-Darcy coefficient and the particle size of sand is also fitted by the exponential function.

## Background

**Fig. 1:**
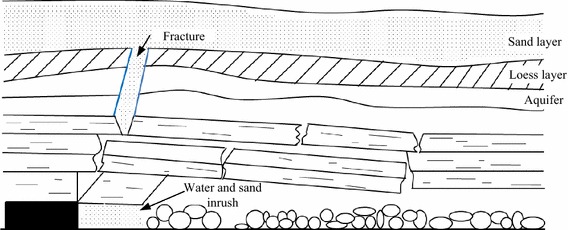
Water and sand inrush in the fracture

In China, water and sand inrush is very serious safety problem for coal mining in 20 years, there were many accidents which gave more damage to coal mining (Limin et al. 2015, 2016). The coal reserves are located at shallow depths and the thin bedrock and thick sand overburdens the strata layers, inducing connected cracks. Surface water, groundwater and sand can flow into the mine goaf through the fractured rock and lead to inrush of water and collapsing of sand, which can be seen in Fig. 1.

From the mechanical perspective, the result of water and sand erupting, permeating fractured rock reflects the instability of the strata layers. Therefore, studying the seepage properties of fractured rocks plays an important role in coal mining engineering. The inrush of water and sand compromises mine safety by causing instability in stress block beams, which creates surface subsidence and water resource run off.

Field tests that are conducted in order to replicate water and sand inrush are difficult; therefore, many scholars suggested conducting experimental simulations of inrushing water and sand. Yang (2009), Yang et al. (2012) and Sui et al. (2007) analyzed the angle of fluid using cemented sand to analyze the mechanisms supporting the inrushing of water and sand. The flow law was examined during various conditions and critical hydraulic gradients of sand inrush currents. Sui et al. (2008) and Xu et al. (2012) analyzed the initial position of inrushing sand based on the structure of water inrush.

Based on underground water dynamic theories, Zhang et al. (2006) created the critical condition and forecasting formula for the prevention of sand inrush by calculating the hydraulic head. Wu (2004) designed a mechanical model of sand inrush pseudo structures, and discussed the force during sand inrush and described the theory of expression of sand inrush. Zhang et al. (2015a) used a case study to discuss drills resulting in sand inrush based on the funnel model. Zhang et al. (2015b) studied the relationship between backfill and water through conducting crack zone. Moreover, river sediment engineering, the theory of sediment transmission and sediment transport mechanics are excellent subject matters to aid in studying the start and movement of sand in mines. Furthermore, the study of sediment engineering, sediment transport theory and practice, and sediment kinematics can aid in understanding the commencement, flow and inrushing sand problem (Du 2014). But others discussed water and sand form the pressure, water and sand flow in tunnel or broken rock (Limin et al. 2016; Du 2014), but the important is seepage in the fracture, which has not been discussed. The concentration and particle’s influence on water and sand inrush.

In this work, permeability attributes of water–sand mixtures are obtained through testing by replicating the design system of water–sand seepage in fractures. The influence of mass concentration in water and particle size of sand on the seepage parameters are tested using specially designed instruments.

## Viscosity test of water and sediment

**Fig. 2:**
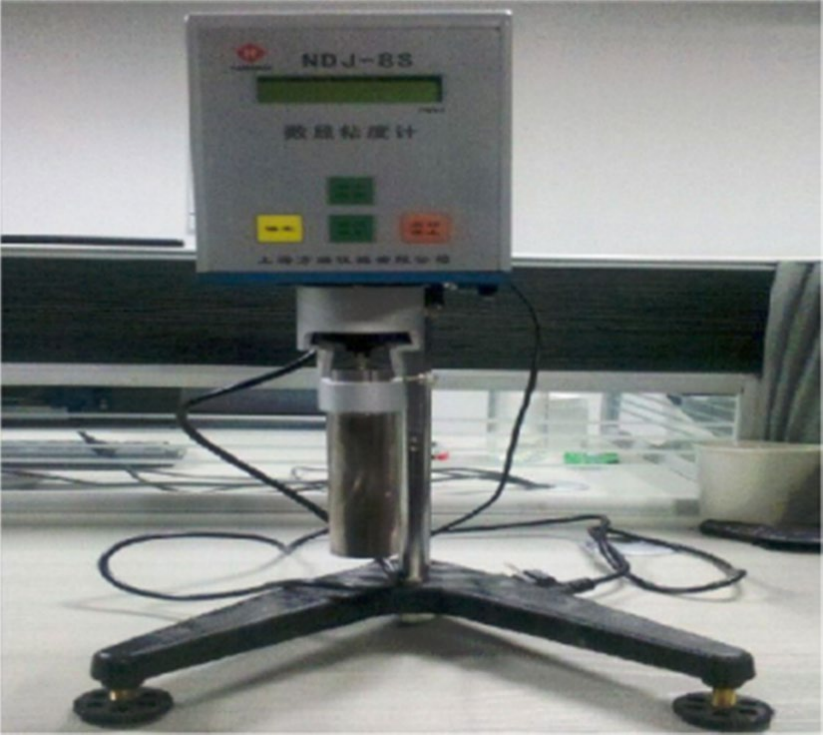
NDJ-8S viscometer

Viscous parameters of water–sand mixture in stress-strain relationships were tested using a NDJ-8S viscosimeter in Fig. 2:

Shear strain rate *γ* of water–sand is defined as

1 $$\gamma = \frac{{\pi n_{rot}^{{}} }}{30} \times \frac{d}{(D - d)}$$

where *D* is the diameter of outer cylinder, *d* is the diameter of the rotor.

Apparent viscosity $\mu_{a}^{{}}$ of water–sand mixture were obtained from the NDJ-8S viscosimeter and the shear stress was calculated as follows

2 $$\tau = \mu_{a}^{{}} \gamma$$

By changing the rotational speed of the NDJ-8S viscosimeter, several values of shear strain rate *γ and* shear stress were obtained and plotted on a $\gamma - \tau$ scatter diagram. According to the shape of the $\gamma - \tau$ diagram, the water–sand mixture was identified as non-Newton fluid, and the viscous parameter of water–sand was obtained through linear regression.

**Fig. 3:**
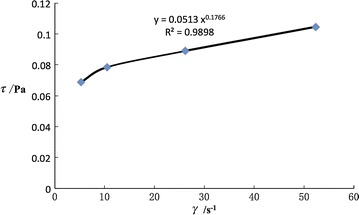
Scatter plot of angle strain rate—shear stress (0.061–0.080 mm)

During the experiment, the diameters of sand particle are 0.038–0.044, 0.061–0.080, 0.090–0.109 and 0.120–0.180 mm. Firstly, Sand particles 0.061–0.080 mm with 20 kg/m^3^ sand at 20 °C was measured; the shear strain rate $\gamma$, apparent viscosity $\mu_{a}^{{}}$ and stress $\tau$ of the water–sand mixture was gotten at various rotation rates (Table 1; Fig. 3).

**Table 1:** Angle strain rate, apparent viscosity and shear stresses at different rotating speed

Rotational speed (rpm)	Shear strain rate (s^−1^)	Apparent viscosity (Pa s)	Shear stress (Pa)
6	5.24	0.0013	0.0687
12	10.47	0.0075	0.0785
30	26.17	0.0034	0.088967
60	52.33	0.002	0.104667

It can be seen that the shear strain rate *γ* increases monotonously along with the shear strain of the water–sand mixture. Therefore, we assume that the water–sand mixture is a power law fluid as follows

3 $$\tau = C\gamma_{{}}^{n}$$

where *C* is the consistency coefficient, *n* is the power exponent.

Combining Eqs. 2 and 3 yields expression of apparent viscosity as follows

4 $$\mu_{a}^{{}} = C\gamma^{n - 1}$$

Through linear regression, viscous parameters of water–sand (consistency coefficient *C* and power exponent *n*) were obtained, as shown in Table 2. It was deduced that the water–sand mixture was a pseudo-plastic fluid, whose viscous parameters changed with sand particle $d_{s}$ and mass concentration of sand $\rho_{s}$.

**Table 2:** Relation of surface viscosity and shearing rate

Particle size (mm)	Concentration (kg/m^3^)	Regression equation	Power exponent	Consistency coefficient (N S^n^/m)
0.038–0.044	20	$\mu_{a} = 0.0569\gamma_{{}}^{ - 0.7613}$	0.2387	0.0569
	40	$\mu_{a} = 0.0617\gamma_{{}}^{ - 0.8046}$	0.1954	0.0617
	60	$\mu_{a} = 0.0623\gamma_{{}}^{ - 0.8401}$	0.1599	0.0623
	80	$\mu_{a} = 0.0701\gamma_{{}}^{ - 0.8692}$	0.1308	0.0701
0.061–0.080	20	$\mu_{a} = 0.0413\gamma_{{}}^{ - 0.8235}$	0.1765	0.0513
	40	$\mu_{a} = 0.5016\gamma_{{}}^{ - 0.8433}$	0.1567	0.0516
	60	$\mu_{a} = 0.0527\gamma_{{}}^{ - 0.8744}$	0.1256	0.0527
	80	$\mu_{a} = 0.0547\gamma_{{}}^{ - 0.9091}$	0.0909	0.0547
0.090–0.109	20	$\mu_{a} = 0.0487\gamma_{{}}^{ - 0.8358}$	0.1642	0.0487
	40	$\mu_{a} = 0.0509\gamma_{{}}^{ - 0.8672}$	0.1328	0.0509
	60	$\mu_{a} = 0.0513\gamma_{{}}^{ - 0.8996}$	0.1004	0.0513
	80	$\mu_{a} = 0.0525\gamma_{{}}^{ - 0.9208}$	0.0792	0.0525
0.120–0.180	20	$\mu_{a} = 0.0416\gamma^{ - 0.9159}$	0.0841	0.0416
	40	$\mu_{a} = 0.0437\gamma_{{}}^{ - 0.9258}$	0.0742	0.0437
	60	$\mu_{a} = 0.0489\gamma_{{}}^{ - 0.9298}$	0.0702	0.0489
	80	$\mu_{a} = 0.0507\gamma_{{}}^{ - 0.9369}$	0.0631	0.0507

Different consistencies were tested of coefficient *C* and power exponent *n* with the diameters of sand particle sizes 0.038–0.044, 0.061–0.080, 0.090–0.109 and 0.120–0.180 mm; and sand 20, 40, 60 and 80 kg/m^3^ in the water. The testing results of consistency coefficient *C* and power exponent *n* are shown in Table 2.

From Table 2, consistency coefficient *C* increases with mass concentration in water as exponential relationship, and decreases along with the increase of sand particle; power exponent *n* increases along with the increase of mass concentration in water, and decreases along with the increase of sand particle.

## Seepage test of water and sand in a fracture

### Test principle

**Fig. 4:**
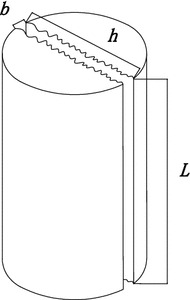
Seepage in parallel fracture

Figure 4 demonstrates a model of seepage in a fracture. In this paper, the aperture of fracture is 0.75 mm, the length is 12.5 mm, the height is 75 mm. *b* is the aperture of fracture, *h* is the height of the fracture, and *L* is the sample length.

According to Fig. 4, we can get Eq. 5.

5 $$V = \frac{Q}{bh}$$

where *V* is the velocity of seepage, *Q* is the flow of seepage.

For the fracture, *Re* is defined as Eq. 6 (Javadi et al. 2014).

6 $$R_{e} = \frac{\rho Q}{\mu b}$$

where *Re* is Reynolds number, *ρ* is the density, *Q* is the flow of seepage, μ is the fluid viscosity.

In the paper, *Q* is $6.00 \times 10^{ - 4} {-}3.10 \times 10^{ - 3}$ m^3^/s, $\rho = 1.02{-}1.08 \times 10^{3}$ kg/m^3^, $\mu = 1.005\;{\text{mp}}_{\text{a}} \;{\text{s}}$.

So, $R_e = \frac{\rho Q}{\mu b} = 76.5{-}421.2$ in case of higher Reynolds numbers ($R_e \gg 1$), the pressure losses pass from a weak inertial to a strong inertial regime, described by the Forchheimer equation (Forchheimer 1901; Chin et al. 2009; Cherubini et al. 2012, 2013; Javadi et al. 2010; Li et al. 2008), given by:

7 $$\rho c_{a} \frac{\partial V}{\partial t} = - \frac{\partial p}{\partial l} - \frac{\mu }{k}V - \rho \beta V^{2}$$

where $\mu$ is fluid viscosity, $\beta$ is non-Darcy factor, the pressure is $p$, $\frac{\partial p}{\partial l}$ is the pressure gradient, $c_{a}$ is the acceleration of water and sand, $b_{1}$ is two term coefficient.

Because of water and sand permeability parameter’s particularity (permeability parameter is relevant to liquid and fracture), we use $\mu_{e}$, $k_{e}^{{}}$ to describe the water and sand of effective viscosity $\mu_{e}$, effective permeability $k_{e}^{{}}$, as shown in Eq. 8 (Liu 2014).

8 $$\rho c_{a}^{{}} \frac{\partial V}{\partial t} = - \frac{\partial p}{\partial l} - \frac{{\mu_{e}^{{}} }}{{k_{e}^{{}} }}V_{{}}^{n} - \rho \beta V_{{}}^{2}$$

As for one kind of non-Newton fluid, liquid viscosity and permeability in fracture of water–sand mixture were related to fluid properties and fracture aperture. Therefore, liquid viscosity and permeability were not obtained separately, and the effective fluidity $I_{e}^{{}}$ was introduced to simplify the expression.

9 $$I_{e}^{{}} = \frac{{k_{e} }}{{\mu_{e} }}$$

The Eq. 8 can be changed into

10 $$\rho c_{a}^{{}} \frac{\partial V}{\partial t} = - \frac{\partial p}{\partial l} - \frac{1}{{I_{e}^{{}} }}V_{{}}^{n} - \beta \rho V_{{}}^{2}$$

Equation 10 calculated the momentum conservation of water–sand seepage in the fracture. For the seepage in Fig. 4, the steady-flow method was selected to measure water–sand seepage in the fracture. Equation 10 can be deduced into Eq. 11,

11 $$\frac{1}{{I_{e} }}V^{n} + \beta \rho V^{2} = - \frac{\partial p}{\partial l}$$

Substituting Eq. 5 into Eq. 11 yields Eq. 12

12 $$- dp = \frac{1}{{I_{e} }}\left( {\frac{Q}{bh}} \right)^{n} dl + \beta \rho \left( {\frac{Q}{bh}} \right)^{2} dl$$

*b* is the aperture of the fracture, *m* is the mass of sand and water.

For the length, the integrating range is [0, *L*]; the mass is *m*, the pressure of water and sand at the entrance wall were:

13 $$\left\{ {\begin{array}{l} {\left. p \right|_{x = 0}^{{}} = p_{0}^{{}} } \\ {\left. p \right|_{x = L}^{{}} = 0} \\ \end{array} } \right.$$

The definite integral of Eq. 12 on the interval [*0, L*] was

14 $$p = \frac{L}{{I_{e} }}\left( {\frac{Q}{bh}} \right)^{n} + \beta mL\left( {\frac{Q}{bh}} \right)^{2}$$

Introducing the sign $\lambda_{1} = \frac{1}{{I_{e} }}\left( {\frac{1}{bh}} \right)^{n}$, $\lambda_{2} = \frac{m\beta }{{(hb)^{2} }}$,

Therefore, Eq. 14 was obtained by using

15 $$\lambda_{1} Q^{n} + \lambda_{2} Q^{2} - p_{0} = 0$$

In the test, 5 flows were set as $Q_{i}^{{}} ,i = 1,2, \ldots ,5$. Steady state values of inlet pressures were tested, and coefficients $\lambda_{1}^{{}}$ and $\lambda_{2}^{{}}$ were fitted. The specific process was as follows:

Equation 15 was obtained

16 $$\varPi = \sum\limits_{i = 1}^{5} {\left( {\lambda_{1}^{{}} Q_{i}^{n} + \lambda_{2}^{{}} Q_{i}^{2} - p_{0}^{i} } \right)_{{}}^{2} } = 0$$

In order to get the least value of the flow *Q*, Eq. 16 can be set as Eq. 17.

17 $$\left\{ \begin{aligned} \left( {\sum\limits_{i = 1}^{5} {Q_{i}^{n} Q_{i}^{n} } } \right)\lambda_{1}^{{}} + \left( {\sum\limits_{i = 1}^{5} {Q_{i}^{2} Q_{i}^{n} } } \right)\lambda_{2}^{{}} = \left( {\sum\limits_{i = 1}^{5} {Q_{i}^{n} p_{0}^{i} } } \right) \hfill \\ \left( {\sum\limits_{i = 1}^{5} {Q_{i}^{2} Q_{i}^{n} } } \right)\lambda_{1}^{{}} + \left( {\sum\limits_{i = 1}^{5} {Q_{i}^{2} Q_{i}^{2} } } \right)\lambda_{2}^{{}} = \left( {\sum\limits_{i = 1}^{5} {Q_{i}^{2} p_{0}^{i} } } \right) \hfill \\ \end{aligned} \right.$$

$\lambda_{1}$ and $\lambda_{2}$ were solved by Eq. 16, effective mobility $I_{e}$ and non-Darcy $\beta$ were obtained.

### Experimental equipment and steps

**Fig. 5:**
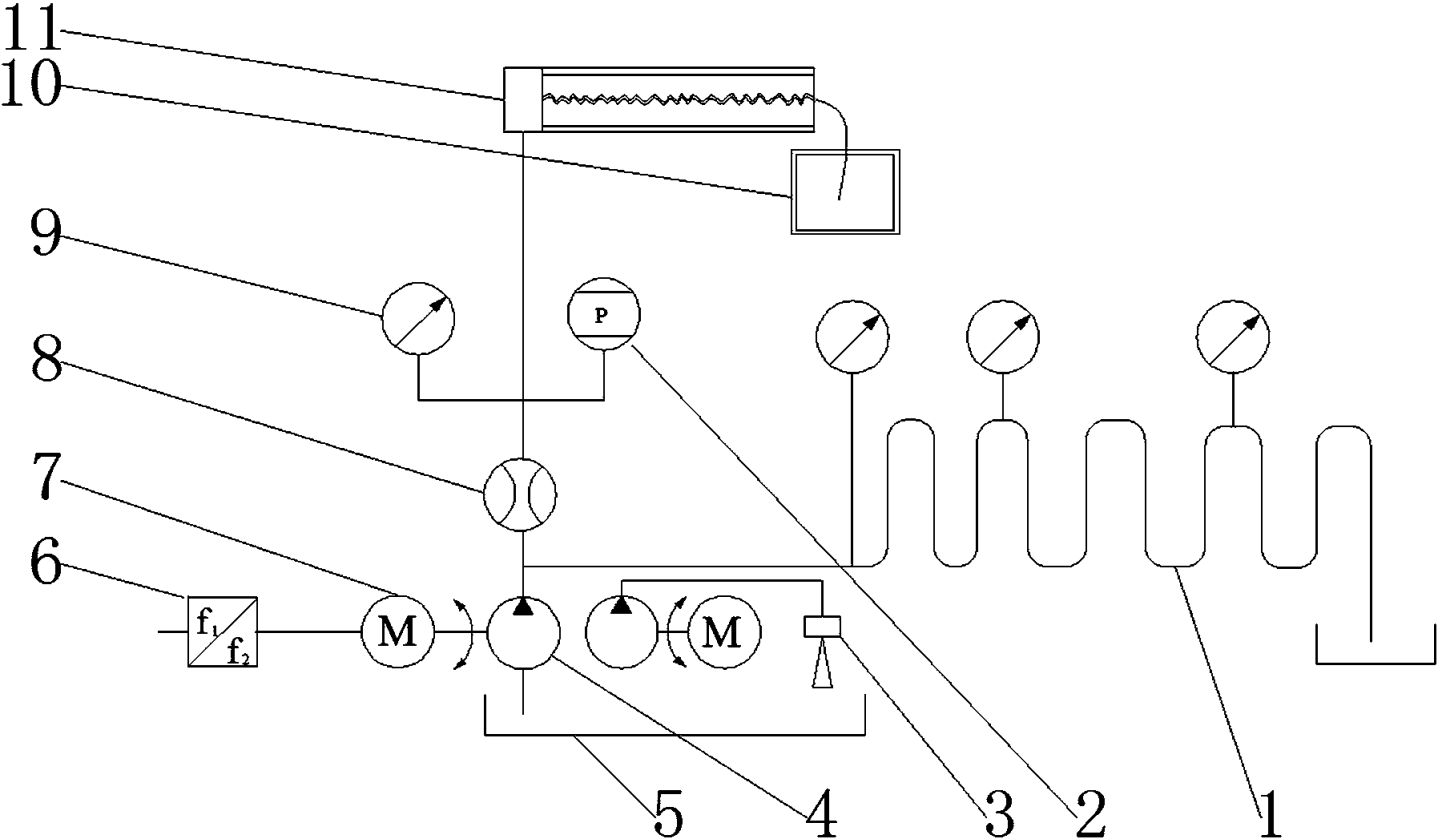
Scheme of system principles. *1* Sidebend; *2* pressure transmitter; *3* injection pipe; *4* vane pump; *5* agitator tank; *6* VVVF; *7* screw pump; *8* flow sensor; *9* piezometer; *10* filter box; *11* scheme of permeameter

Based on testing principles, a set of experimental system was designed and manufactured as shown in Fig. 5. Sand comes from the surface of the mine in northwest of China. The rock sample is the sandstone under −265 m from the Luan mine in Shanxi, China. There are five specimens of rock fracture with Joint Roughness Coefficient (*JRC*) 4–6, the velocity of seepage was obtained.

**Fig. 6:**
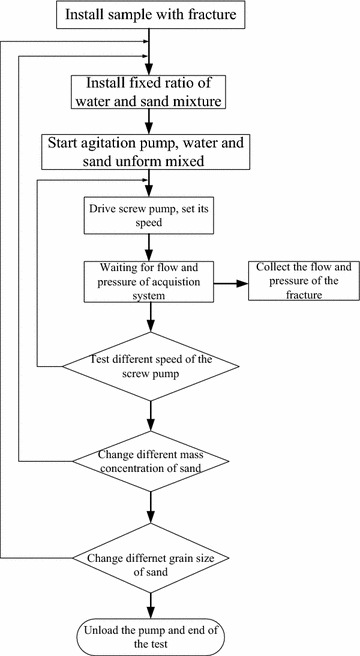
Flow chart of the test

Figure 6 illustrates the entire experimental procedure. The test steps were as follows:

1. The test system was assembled according to Fig. 6 and the sample was loaded. The leakage of the experiment system was tested.
2. The sand grain with a diameter of 0.038–0.044 mm was placed into the mixing pool and the sand concentration was 20 kg/m^3^ in water.
3. To control the motor speed, flow and pressure under different rotational speeds were recorded while the fracture aperture 0.75 mm; the motor speeds, 200, 400, 600, 800, 1000 r/min were changed separately. Different pressures and seepage velocities of the fracture were obtained using a paperless recorder. The sand concentration $\rho_{s}$ in water was 40, 60, 80 kg/m^3^ respectively.
4. The flow and pressure under different grain diameters (0.038–0.044, 0.061–0.080, 0.090–0.109 and 0.120–0.180 mm)were recorded during the different rotational speeds. In order to easily calculate the data, we choose the arithmetic mean of each range of the grain diameter, e.g. 0.041, 0.071, 0.100 and 0.150 mm.
5. According to Eqs. 15 and 16, $I_{e}$ and $\beta$ were calculated.

## Results

### Pressure graduate

**Fig. 7:**
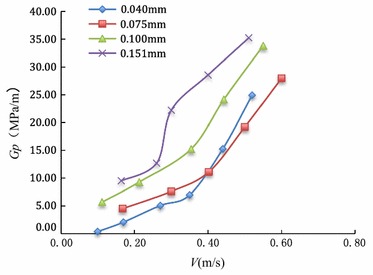
Relationship between pressure gradient and velocity

According to the pressure and velocity measured in the tests, the pressure gradient and velocity under different sand concentration in water were shown in Fig. 7 and Table 3. Table 3 lists the test result of pressure gradient and seepage velocity of the water and sand, the polynomial fitting formula and its coefficient, the power fitting formula and its coefficient.

**Table 3:** Relationship between pressure gradient and velocity under different sand concentration

Number	Concentration (kg/m^3^)	Pressure gradient (MPa/m)	Velocity (m/s)	Polynomial function	Power function
1	20	0.38	0.10	$G_{p} = 155.61V^{2} - \, 41.24V + \, 3.72$ R^2^ = 0.9882	$G_{p} = 0.29e^{9.04V}$ R^2^ = 0.9259
		2.10	0.17		
		5.08	0.27		
		6.98	0.35		
		15.27	0.44		
		24.92	0.52		
		4.54	0.17		
2	40	7.62	0.30	$G_{p} = 118.08V^{2} - \, 36.42V \, + \, 7.43$ R^2^ = 0.9970	$G_{p} = 2.14e^{4.28V}$ R^2^ = 0.9954
		11.11	0.40		
		19.21	0.50		
		27.98	0.60		
		5.71	0.11		
3	60	9.30	0.26	$G_{p} = 108.12V^{2} - \, 7.42V \, + 5.38$ R^2^ = 0.9956	$G_{p} = 3.74e^{4.07V}$ R^2^ = 0.9953
		15.24	0.35		
		24.16	0.40		
		33.81	0.51		
		7.94	0.16		
		12.70	0.27		
4	80	22.22	0.35	$G_{p} = 55.04V^{2} + 119.60V - 11.08$ R^2^ = 0.9602	$G_{p} = 4.50e^{4.35V}$ R^2^ = 0.8968
		28.57	0.44		
		35.24	0.51		

Figure 7 and Table 3 been presented above, we can obtain: the seepage velocity of water and sand increases with pressure gradient increasing, Moreover, the greater the sand concentration in water is, the lower the seepage velocity is.

### Permeability of water and sand in the fracture

**Fig. 8:**
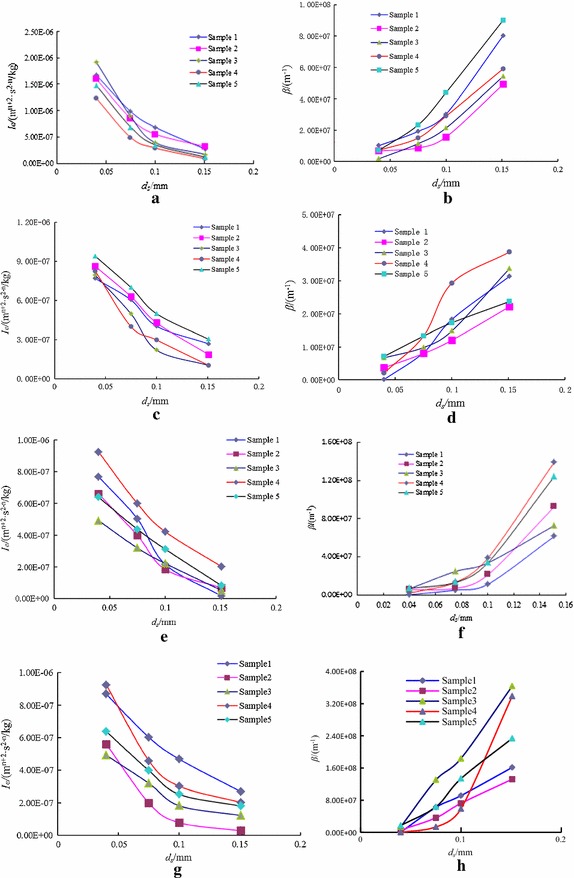
Curves of permeability parameters changing with $d_{s}$. **a** Curve of $I_{e} - d_{s}$ under 20 kg/m^3^, **b** curve of $\beta - d_{s}$ under 20 kg/m^3^, **c** curve of $I_{e} - d_{s}$ under 40 kg/m^3^, **d** curve of $\beta - d_{s}$ under 40 kg/m^3^, **e** curve of $I_{e} - d_{s}$ under 60 kg/m^3^, **f** curve of $\beta - d_{s}$ under 60 kg/m^3^, **g** curve of $I_{e} - d_{s}$ under 80 kg/m^3^, **h** curve of $\beta - d_{s}$ under 80 kg/m^3^

Keeping the fracture aperture 0.75 mm, the permeability parameters of water and sand seepage in the fracture under particle sizes 0.041, 0.071, 0.100 and 0.150 mm are tested at 20, 40 and 60 kg/m^3^ sand concentration in water, as shown in Fig. 8, liquid viscosity and permeability were not obtained separately, and the effective fluidity $I_{e}^{{}}$ was introduced to simplify the expression.

Because of the permeability parameters of water and sand seepage in fracture are connected with water and sand, at the same time, the structure of fracture; so the permeability *k* is not enough to describe permeability parameters, the effective fluidity and non-Darcy factor $\beta$ are used.

The 5 samples were used to obtain the permeability parameters in test, and we adopted the arithmetic mean values, as shown in Table 4.

**Table 4:** permeability parameters of water and sand under different sand concentration

Concentration of sediment (kg/m^3^)	Particles size	$I_{e}$ $I_{e} \;\left( {{\text{m}}_{{}}^{n + 2} \cdot {\text{s}}_{{}}^{2 - n} /{\text{kg}}} \right)$	$\beta \;({\text{m}}_{{}}^{ - 1} )$
20	0.04	1.57E−06	5.67E+06
	0.075	7.62E−07	1.46E+07
	0.100	4.34E−07	2.64E+07
	0.151	1.71E−07	6.48E+07
40	0.04	8.34E−07	2.14E+06
	0.075	5.53E−07	1.03E+07
	0.100	3.54E−07	1.75E+07
	0.151	1.73E−07	2.93E+07
60	0.04	6.82E−07	3.04E+06
	0.075	4.41E−07	1.10E+07
	0.100	2.60E−07	2.54E+07
	0.151	6.31E−08	9.33E+07
80	0.04	6.76E−07	1.05E+06
	0.075	6.76E−07	4.77E+07
	0.100	2.20E−07	9.90E+07
	0.151	1.28E−07	2.28E+08

Fitting the curves of Fig. 8, the functional relationship between seepage parameters and sand concentration in water was used, as shown in Table 5.

**Table 5:** Fitted equations of permeability parameters changing with $d_{s}$ at *JRC* 4–6

Number	Sand concentration (kg/m^3^)	Permeability parameters	Fitting equations	Coefficient
1	20	$I_{e}$	$I_{e} = 3.40 \times 10^{ - 6} e^{{ - 20.03d_{s} }}$	0.9980
		$\beta$	$\beta = 2.64 \times 10^{6} e^{{21.79d_{s} }}$	0.9872
2	40	$I_{e}$	$I_{e} = 1.65 \times 10^{ - 6} e^{{ - 14.80d_{s} }}$	0.9693
		$\beta$	$\beta = 3.10 \times 10^{5} e^{{33.82d_{s} }}$	0.7805
3	60	$I_{e}$	$I_{e} = 1.95 \times 10^{ - 6} e^{{ - 21.81d_{s} }}$	0.9688
		$\beta$	$\beta = 1.01 \times 10^{6} e^{{30.66d_{s} }}$	0.9905
4	80	$I_{e}$	$I_{e} = 1.15 \times 10^{ - 6} e^{{ - 15.10d_{s} }}$	0.9781
		$\beta$	$\beta = 1.78 \times 10^{5} e^{{48.84d_{s} }}$	0.9549

The exponential function was used to fit the relationship between effective fluidity, non-Darcy coefficient and particle sizes of sand. The power exponent equations are used to fit the relationship between effective fluidity *I*_*e*_, the non-Darcy factor $\beta$ and Sand concentration.

From Fig. 8 and Table 5, the following results were obtained:

1. The seepage of water and sand in a fracture is nonlinear.
2. Along with the change of grain size of sediment, the relationship between effective fluidity $I_{e}$ and mass concentration of sand $d_{s}$ was the negative exponential relationship; the absolute value of the exponent increased along with the increase of sand particle in the water.
3. Non-Darcy factor *β* and sand concentration in water had a positive exponential relationship; the absolute value of the exponent increased along with the decrease of sand particle in water.

## Discussion

It is non-Darcy flow in the paper, which was influenced by roughness, flow velocity, aperture of fracture, and so on. Roughness has a large influence on fracture flow, where non-Darcy also happened (Boutt et al. 2006; Lomize 1951; Louis 1969; Qian et al. 2011).

During the flow, Reynolds number and Forchheimer’s number are important parameters to judge (Bear 1972): when *Re* > 100 or *Re* < 1, it will be nonlinear flow and does not conform to Darcy flow. What’s more, the velocity of water and sand, the aperture of fracture and the tortuosity of fracture also have much influence on flow parameters (Tsang 1984; Tsang and Tsang 1987). The concentration and density also have influence on flow character in fracture (Watson et al. 2002; Tenchine and Gouze 2005). Here $I_{e}$ has relationship with the structure of fracture, and the character of mixture or water and sand. With the pressure drop increasing, the nonlinear flow became obvious (Elsworth and Doe 1986; Wen et al. 2006; Yeo and Ge 2001) the Forchheimer’s law is well known classical approach to describe the nonlinear flow in fracture. Non-Darcy factor *β* is the parameter which reflected the deviation of Darcy of the seepage. Along with sand particle in water, the non-Darcy character became more obvious.

## Conclusion

In this paper, the viscosity of water and sand mixture was discussed and the seepage of water and sand mixture in rude fracture was analyzed.

1. The seepage velocity of water and sand in a fracture increases along with the pressure of the fracture, but the relationship between them is nonlinear.
2. Consistency coefficient *C* becomes larger in conjunction with the mass concentration in water, but decreases along with the particle size of sand. The lower exponent *n* becomes enlarger along with mass concentration in water, but decreases along with particle size of sand.
3. Along with the change of the grain size of sediment, the relationship between effective fluidity $I_{e}$ and mass concentration of sediment $\rho_{s}$ in water is exponential. The absolute value of the exponent increases along with the increase of sand concentration in water. The non-Darcy factor *β* and sand concentration in water has a positive exponential relationship and the absolute value of the exponent increases along with the decrease of sand concentration in water.
4. For the future work, we will work for the different concentration, for particle and concentration both has influence to the flow character, but we should do some experiments to make sure which one is more influence. And acceleration, low velocity of water and sand how to change into water and sand inrush.

## List of symbols

*b*: aperture of fracture
*C*: consistency coefficient
*C*_*a*_: acceleration coefficient
*d*: diameter of the rotor
*D*: diameter of outer cylinder
*d*_*s*_: particle size of sand
*h*: height of the fracture
*I*_*e*_: effective fluidity
*k*_*e*_: effective permeability
*L*: sample length
*m*: mass of sand and water
*n*: power exponent
*n*_*rot*_: rotate speed of rotor
*β*: non-Darcy coefficient
*p*: pressure
*Q*: flow of seepage
$$\tau$$: shear stress
$$\mu$$: fluid viscosity
$\mu_{a}$: apparent viscosity of water and sand
$$\mu_{e}$$: effective viscosity
*V*: velocity of seepage
$$\gamma$$: apparent viscosity
$$\rho$$: density
$\rho_{s}$: mass concentration of sand
$$\frac{\partial p}{\partial l}$$: pressure gradient
